# The ethical issues regarding consent to clinical trials with pre-term or sick neonates: a systematic review (framework synthesis) of the analytical (theoretical/philosophical) research

**DOI:** 10.1186/s13063-016-1562-3

**Published:** 2016-09-09

**Authors:** Christopher Megone, Eleanor Wilman, Sandy Oliver, Lelia Duley, Gill Gyte, Judy Wright

**Affiliations:** 1 Inter-Disciplinary Ethics Applied, University of Leeds, Leeds, UK; 2 Public Policy, Social Science Research Unit, EPPI-Centre, UCL Institute of Education, London, UK; 3 Clinical Trials Research, Nottingham Clinical Trials Unit, Nottingham Health Science Partners, University of Nottingham, Nottingham, UK; 4 National Childbirth Trust, London, UK; 5 Leeds Institute of Health Sciences, University of Leeds, Leeds, UK

**Keywords:** Research, Ethics, Pre-term/sick neonates, Parents, Clinicians, Consent, Validity, Competence, Voluntariness, Information, Risk

## Abstract

**Background:**

Conducting clinical trials with pre-term or sick infants is important if care for this population is to be underpinned by sound evidence. Yet, approaching the parents of these infants at such a difficult time raises challenges to obtaining valid informed consent for such research. In this study, we asked, What light does the analytical literature cast on an ethically defensible approach to obtaining informed consent in perinatal clinical trials?

**Methods:**

In a systematic search, we identified 30 studies. We began our analysis by applying philosophical frameworks, which were then refined as concepts emerged from the analytical studies, to present a coherent picture of a broad literature.

**Results:**

Between them, the studies addressed four themes. The first three were the ethical basis for parental informed consent for neonatal and/or perinatal research, the validity of parental consent in this context, and the range of possible options in methods for gaining consent. The last was the issue of risk and the possibility of a double-standard or asymmetry in the current approaches to the requirement for consent for research and consent for clinical treatment.

**Conclusions:**

In addressing these issues, the analysed studies showed that, whilst there are a variety of possible defences for seeking parental ‘consent’ to neonatal and/or perinatal clinical trials, these are all consistent with the strongly and widely held view that it is important that parents do give (or decline) consent for such research. So far as the method of obtaining consent is concerned, none of the existing consent processes reviewed by the research is satisfactory, and there are philosophical reasons for supposing that at least some parents will fail to give valid consent in a neonatal context. Furthermore, in giving parental ‘consent’ in a perinatal context, parents are authorising infant participation, not giving ‘proxy consent’. Finally, there are reasons for giving weight to both parental ‘consent’ and the infant’s best interests in both research and clinical treatment. However, there are also reasons to treat these factors differently in the two contexts, and this may be partly due to the differing relevance of risk in each case. A significant gap is the lack of any detailed discussion of a process of emergency and/or urgent ‘assent’, in which parents assent or refuse their baby’s participation as best they can during the emergency and later give full consent to continuing participation and follow-up.

**Electronic supplementary material:**

The online version of this article (doi:10.1186/s13063-016-1562-3) contains supplementary material, which is available to authorized users.

## Background

The recruitment of pre-term or sick infants to trials requires approaching parents at a particularly difficult time, often within a tight time frame for making a decision. This raises challenges for the obtaining of valid informed consent to such research. But given the importance that has been attached by codes of ethics to gaining informed consent for clinical research, if the problem of consent cannot be successfully addressed, there is a risk of this becoming an ‘orphan’ area of research. That is, if ethically permissible research cannot be designed, then this area of medical research will be abandoned.

This paper reports on analytical research examining and addressing these difficulties. We undertook two systematic reviews of the research addressing these ethical challenges within the context of a larger project focused on improving care for the pre-term infant. These reviews had as their goal the identification of both the ethical challenges and potential solutions. The aim of the review of the empirical research was to synthesise observational and qualitative studies that explored the process of recruitment and consent, and parents’ and clinicians’ views and experiences of that process [1].

The present review is a complementary paper whose aim is to synthesise analytical (or philosophical) studies which have examined the pertinent ethical questions. These concern the validity of consent, the proper understanding of the parental role in giving or withholding consent, varied possible methods of seeking consent, the best interests of those involved, issues of risk, and the parallels between consent processes in relevant research and clinical contexts.

The review process for the two papers, empirical and analytical, involved a common literature search and common elements in the development of the conceptual framework which both guided that search and was informed by its results. The process then involved initially identifying papers found in the search as empirical or analytical and, once that initial distinction had been made, proceeding in two separate processes of identifying more precisely relevant themes and then coding papers. In the light of the common features in the development of these two reviews, there is inevitably some overlap between this analytical paper and the empirical paper [1], both in setting out the broad context and in describing the method for the research.

The use of a systematic review for addressing problems in medical ethics is methodologically novel, perhaps particularly so with respect to the treatment of analytical papers [2–4]. In philosophy, the standard way to engage with analytical papers is to subject their arguments to scrutiny rather than to synthesise their main claims. The method of systematic review as developed in the social and medical sciences is designed, it would seem, to achieve objectivity and impartiality in arriving at a synthesis of results of research in a given field. Philosophers might claim that the method of analysing and developing arguments achieves a similar objectivity and lack of bias in virtue of the impartial nature of the analysis and development of arguments. Having said that, there is a methodological tradition in philosophy, going back to the Greeks [5, 6], which suggests that, in any inquiry, the appropriate place to start is by reviewing the ‘*phainomena*’, and these *phainomena* include the existing views of both the ordinary person and the wise on the topic in question. Given the very large output of analytical papers in contemporary research, such a comprehensive review of the *phainomena* is perhaps less common and would arguably be hard to fulfil on some topics. So it might be said that the standard method of analytical engagement with analytical papers does run the risk of considering only a narrow perspective on an issue. Thus, to that extent, it also runs the risk of a certain sort of bias, a bias that the methods adopted in a systematic review do help to avoid.

The aim of the present paper is to synthesise the key strands of philosophical argument that have been put forward with respect to consent in neonatal research, rather than to engage dialectically with them. For clinicians, the key ethical question is practical: “What is the right thing to do here?” That is a central ethical question, but it is also important for clinicians to ask why a course of action is right, and the analytical literature is focused on that.

Our research has been undertaken by a multi-disciplinary team of philosophers, clinicians, social scientists and information scientists who have brought complementary skills to the task. So, this process of developing a systematic review in ethics through inter-disciplinary collaboration has given rise to considerable reflection in its own right. These unpublished observations of authors of this paper are being collated. However, in this paper, no significant space is given to detailed further reflection on that process.

The paper begins by setting out a brief summary of an iterative process for making sense of a body of literature via a framework synthesis, then gives an account of the results, and concludes with a discussion of some of the key points arising from the results. Our overarching question was, What light does the analytical literature cast on an ethically defensible approach to obtaining informed consent in perinatal research?

In broad outline, the method adopted for this review conformed to that set out for a framework synthesis in Gough et al.’s recent account of systematic reviews [7]. The first stage was the development of a tentative initial conceptual framework^1^ which relied mainly on prior knowledge of the existing philosophical literature on informed consent (prominent books and papers). This included knowledge of material that was more specifically focused on the difficulties of gaining informed consent in neonatal research [8, 9]. This initial conceptual framework informed the criteria for including studies and suggested terms for the first literature searches.

The initial conceptual framework was then refined in light of the literature uncovered by the first searches (stage 2 in Gough et al.’s account [7]) and the multiple perspectives offered by the backgrounds of the authors. The second searches were then developed, informed by the now-confirmed conceptual framework. Searches were run on a wider set of resources to address the multidisciplinary nature of the review. At stage 3, the task of coding articles was undertaken, and this led to stage 4, the tabulating of the data. The final stage was then drawing conclusions from the tabulated data (stage 5).

## Methods

The aim of the searches was to identify studies for a review of ethical issues around consent that arise from the involvement of either pre-term babies or sick neonates in clinical trials. The first literature searches were undertaken in July 2011 in MEDLINE (Ovid) and Philosopher’s Index (Ovid). The terms and synonyms for the search were informed by the initial conceptual framework and the project team’s suggestions. The information specialist identified subject headings and constructed search strategies by combining search terms and subject headings.

Articles potentially eligible for inclusion were those reporting consent and decision-making in various perinatal or related contexts:

1. Any empirical research or analysis and/or commentary concerning the following:
  - Consent, participation or recruitment for neonatal research
  - Parental decision-making for treatment of, or research with, sick or pre-term neonates
  - Parental decision-making for birth and/or labour
  - Methodology in emergency and/or urgent research
  - Alternative ways of gaining consent
2. Full text available in English

Titles and abstracts, and subsequently full texts, were retained only if they matched the inclusion criteria. Articles were screened by a researcher with clinical and ethics expertise (EW) who adopted an overinclusive approach to avoid losing any relevant studies; in case of any doubt, the decision was discussed with a philosopher (CM).

The ‘included’ articles were then separated into ‘empirical’ (quantitative or qualitative research) and ‘analytical’ (philosophical discussion or commentary) categories. The ‘analytical’ papers which were identified as potentially relevant after screening the output of the first systematic search were re-read to identify recurrent themes. Draft summaries of each theme were then distributed to the steering group, which comprised a social scientist, another clinician, a lay representative and the information specialist. They confirmed the conceptual framework.

Further searches were devised with attention to the confirmed conceptual framework. These second searches were conducted in October 2011 on 11 databases (see Additional file 1). The aim of these searches was to identify studies of relevance to the review themes despite coming from different disciplines (e.g., obstetrics, foetal-maternal medicine, neonatology, social science, bioethics). The additional databases were chosen on the basis of their availability and the advice offered in the literature on bioethics studies retrieval [3, 4, 10, 11]. The search terms were informed by the confirmed conceptual framework and by terms suggested in bioethics review methods studies [3, 4, 10]. Update searches were conducted in January 2014 in all previously chosen databases. Additional file 1 shows an example search strategy.

The results of the second searches were then considered. Papers from these references were screened, and ‘included’ or ‘excluded’, on the basis of abstracts using the inclusion criteria set out above. However, in this second screening, the review team demanded, in addition, very definite positive reasons to include further papers. There had to be clear indications that some new insights were being added to those we already had from the initial set of papers. In the absence of such new insights, the research findings were viewed as saturated. In the next stage (stage 3), the findings of these included analytical papers were coded according to the confirmed conceptual framework, including emergent themes, and tabulated to provide a summative identification of the key ethical points made in the papers analysed.

## Results

The Preferred Reporting Items for Systematic Reviews and Meta-Analyses (PRISMA) diagram (Additional file 2) illustrates the flow of studies throughout the review. In the searches, we identified 1361 records (234 from the first searches, 790 from the second round and 327 from the update searches). A set of 27 papers identified from the first searches met the original inclusion criteria. Expanding the databases and introducing new search terms while narrowing the scope (second searches) led to a total of 30 analytical papers being ‘included’. Recurrent themes were identified in the literature (Table 1).

**Table 1:** Recurrent themes and their elaborations

Theme	Elaboration
The ethical basis of parental informed consent for neonatal research	The ethical reasons put forward for the importance of gaining parental consent for neonatal research
Validity of parental consent	Discussion of the requirements to be met if parental consent given is to be viewed as genuine informed consent
Other options for gaining consent	Discussion of different possible methods for gaining consent
Risk and the double-standard between consent for treatment and consent for research	The ethically defensible level of risk to which a neonate undertaking research might be exposed and comparisons with the approach to risk and consent in treatment

Table 2 presents the themes covered by papers included in the review and the context in which the arguments were developed. The table includes only the papers which were first to identify a unique theme and research context. Subsequent papers are not shown, as Table 2 displays the themes present within different research contexts and does not quantify the number of papers per theme and context. However, all the papers are referred to in the results prose and in the tabulated results (Additional file 3).

**Table 2:** Themes and context of the philosophical arguments

		Themes revealed				
		Ethical basis for consent	Validity of consent	Issues of risk	Other options for consent	Miscellaneous topics
Context in which philosophical arguments have been developed	Emergency neonatal research		Manning, 2000 [24]		Manning, 2000 [24]	
	Perinatal clinical decision-making	Pinkerton et al., 1997 [20]				
	Foetal-maternal research		American College of Obstetricians and Gynecologists, Committee on Ethics; American Academy of Pediatrics, Committee on Bioethics, 2011 [26]			American College of Obstetricians and Gynecologists, Committee on Ethics; American Academy of Pediatrics, Committee on Bioethics, 2011 [26]
	Neonatal clinical decisions	Rostain and Bhutani, 1989 [13]	Rostain and Bhutani, 1989 [13]	Rostain and Bhutani, 1989 [13]		
		Schneider, 1988 [15]				
	Neonatal research	McDonnell, 1990 [14]	Silverman, 1988 [29]	Silverman, 1988 [29]	Silverman and Altman, 1996 [35]	
				Silverman and Altman, 1996 [35]	Tyson and Knudson, 2000 [32]	
				Tyson and Knudson, 2000 [32]	Allmark, 1999 [40]	
				Vain et al., 2004 [38]		
	Perinatal emergency research				Vain et al., 2004 [31]	
	Trials in general (but discusses ECMO)				Worrall, 2008 [39]	
					Braunholtz, 1999 [41]	

*ECMO* Extracorporeal membrane oxygenation

In what follows, first of all, we present these tabulated results in prose form, and then, in the last section, we discuss the conclusions drawn from those results.

### The ethical basis for (parental) informed consent in neonatal and/or perinatal research

The focus of the analytical papers which addressed the issue of parental informed consent in the neonatal and/or perinatal context was on the question of the nature of the justification for seeking consent from a parent (or parents). This issue arises because it may be thought that it is not sensible to seek consent when the most affected party is unable to give consent. It might be suggested that the focus should simply be on that individual’s best interests. This approach might be buttressed by querying the basis for consent being given by another, in this case the parent.

So, one claim is that, where children are unable to give or decline consent, parents should do so. A number of justifications are offered for this. Some are based on an appeal to the importance of autonomy. Different forms of the appeal to autonomy are then made.

One view put forward appeals to the importance of parental autonomy, or the parents’ own rights, in this context [12, 13]. Thus, it is held that making decisions about a child is part of what it is to be a parent [14] or, alternatively (or in addition), that respecting parental autonomy here is part of respecting a ‘privacy’ right of the parent [15]. A similar but slightly different claim holds that parenting decisions are part of deciding how to conduct one’s own life [13, 15], whilst another defence suggests that foetal rights are a function of maternal autonomy [16].

However, others have rejected any defence of parental consent that appeals to autonomy. They have objected that a parent’s interest in autonomous parenting may not outweigh the child’s interests [15], or that parents are no longer ‘owners’ of their children [17].

A second claim in defence of parental consent is that parents should be seen as surrogate or proxy decision makers [13]. Thus, it has been suggested that when a child is not yet autonomous, the parent is the most appropriate ‘proxy’ [18], where a ‘proxy’ means not simply someone who acts on behalf of another but one who represents another’s views. A general defence for this is that the parent may be in the best position to have knowledge of the child’s personality, values and beliefs, but this claim is noted to apply only to the case of older children, not to that of neonates [18].

Some suggest there might be reason for a parent to give consent even if an appeal to autonomy or rights is rejected. Thus, it is held that (1) it is not appropriate to think of the ‘autonomy’ of a neonate [17, 18], and therefore (2) it is not appropriate to think of ‘informed consent’ for a child [14].

Another line of argument is that consent in the neonatal context should be seen as a case of ‘family decision-making’, because it is not appropriate to consider the child’s decision in isolation [14]. In addition, it is suggested that parents should give consent because parents will bear the consequences of the decision [13].

However, some claim that the requirement for parental consent rests on the value of beneficence. Thus, the purpose of informed consent is said to be the protection of the best interests of the child, and it is the responsibility of parents to make decisions as a way of promoting their child’s best interests [13, 19], so they should give consent. Or it is said that parents are most likely to have the child’s best interests at heart [15, 20], or that parents are the best judges of their child’s best interests [15, 17, 18, 20]. (Some object to this, that such a justification is prudential and therefore insecure; that is, it is suggested that, in the often traumatic context in which parents are consulted about neonatal research, they often may not be the best judge of their child’s interests [15].) In a related but slightly different vein, some have argued that parents should only be *allowed* to make the decision when it *is* in the best interests of their child [13, 14, 17, 20].

In reply to this, it is argued that the protection of the child’s best interests should not rest entirely or even mainly on informed consent [12, 17]. It is the responsibility of the researcher or the ethical review process to protect the subject’s best interests (so it is inappropriate to defend parental consent by an appeal to the protection of those interests). Others have claimed that determining what is meant by the best interests of a neonate is not simple. There are different accounts of best interests—the mental state account, the desire satisfaction account, and the objective list view. Even if the right account as between these is agreed, the application of at least some such accounts to a neonate is challenging [21].

A further general response to the parental consent view notes that the concept of parental consent is a misnomer here. In the case of neonates, what should be discussed is the concept of parental permission (or authorisation) [17].

### The validity of consent

As in the empirical research we examined, an important theme in the analytical research is the question whether, in the neonatal and perinatal context, parental consent is valid, and to what extent the validity of the consent matters. One aspect of this is the potential barriers to obtaining valid consent in these circumstances [22]. One factor noted is the time limitations that will regularly occur. Such a time limit will adversely affect the amount and quality of the information given [23, 24], and, correspondingly, a time limit will harm the parental understanding of any information given [24].

A second point addresses the stress, anxiety or pain of the parents, as well as the mother’s sedation [23, 25]. It is argued that this can adversely affect understanding [23–25] or capacity [23, 25]. However, some writers have claimed that these features can also contribute (positively) to an autonomous decision process [13].

A third impediment to valid consent that has been identified is the potential desperation or fear of the parents [23, 24, 26]. These emotional states will affect the voluntariness of any consent given (presumably through acting as forms of psychological coercion) [24, 25, 27]. A fourth line of argument is that the researcher may very often be seen by parents as a figure of authority or power, and that this also has a coercive effect on the parents which affects the voluntariness of the decision [28]. A slightly similar argument claims that the consent process itself, through undermining the individuals’ appreciation of their own competence, is an indirect obstacle to autonomy and thus to genuine consent [25]. Another line of argument which identifies a more indirect problem for gaining valid consent is that the consent process itself increases parental anxiety (and thus affects valid consent) [24].

Discussions of this theme also consider the implications of the existence of barriers to informed consent in the context of neonatal or perinatal research. Some address the implications of these barriers for respect for the principle of autonomy if, in light of the discussions identified in the first theme, that is the basis for seeking parental consent. Some argue that parental autonomy will be violated if there is a defect in the consent process [24], while others adopt a Kantian perspective and suggest that parents are being used as a means to an end (not as a member of the ‘Kingdom of Ends’, in Kant’s terms) in a consent process which is flawed in any of the ways identified above [24].

A further argument, building on these problems, is that the principle of beneficence (acting for the benefit of the child and/or mother) becomes a more important ethical principle in research with neonates *because* informed consent is not possible (and therefore any appeal to autonomy or respect for persons should be applied with caution) [27]. However, another response to the difficulties with and barriers to consent is that we must strive for improvements in what we do to seek consent and must attempt to get the best possible consent even where perfect informed consent is not possible [17]. But one worry raised about this way of proceeding is that the residual difficulties will make it the case that deprived or uneducated parents will be more likely to consent. This will therefore mean, it is claimed, both that the practice of neonatal research involves an unjust allocation of any burdens which such research involves and that, from a methodological point of view, any conclusions drawn from the research may not be generalisable [24, 29].

### Issues of risk in neonatal and perinatal research and the possibility of double-standards, or disanalogies, or asymmetries, between consent for research and consent for clinical treatment

The analytical research has also examined a cluster of issues around the treatment of risk in medical research and clinical treatment, and somewhat related matters about differences or parallels in the way in which decisions are reached in clinical and research contexts. Although these are more general issues, they all bear directly on the treatment of risk, consent and best interests in neonatal and perinatal research.

There are disagreements about the nature of the risks involved in research. One claim is that if the context for the clinical trial is a potentially life-threatening condition for the subject, and the outcome of the intervention is unknown, then the trial treatment or intervention should itself be viewed as a significant risk for the subject [30]. An opposing claim is that if the context for the trial is a potentially life-threatening condition, then this is a risk of the disease and/or the situation, and not of the trial, so it should not be viewed as a risk of the trial intervention [31]. A third, somewhat related claim about risk in trials is that fully informed consent is not possible for clinical trials because the very information needed for fully informed consent is that which is uncertain and under investigation [32].

There are then a series of claims as to the parallels between the two areas of practice, or their absence, and these may in part be to do with the evaluation of risk in each context. Thus, it is claimed that in clinical decisions the principle of beneficence tends to trump other ethical considerations, but in research decisions the principle of autonomy trumps considerations of interest [33]. This is conjectured to hold firstly because the two types of consent process evolved separately [29] (with the attitude to consent in research developing through the principles of Helsinki which govern the ethics of research) and secondly because the requirement for the research community to be in a state of equipoise when conducting trials means that appeals to the principle of beneficence are less convincing in a research context [13, 16, 17].

Perhaps following from this last point, it has been argued that where there is genuine equipoise as between the existing and trial treatments, then therapeutic research may not be significantly different, from an ethical point of view, from clinical treatment [33]. If this is so, then it is concluded that there should be no difference in the nature of the ethical principles governing the two practices [32, 33].

Likewise, it has been questioned whether, if there is in fact an inclusion benefit for subjects who participate in trials, research should be viewed as any different from treatment [34]. However, it has also been claimed that the stricter ethical guidelines in research correctly acknowledge the altered doctor-patient relationship when the subject is both a patient and a trial participant [34].

Finally, reverting more directly to issues of risk, it has been claimed that randomised controlled trials are needed specifically to protect patients from the use of untested treatments—in other words, to protect them from the risks associated with those treatments [23, 35]. It has also been held that using unproven therapies in treatment would in itself be a violation of patient autonomy, presumably because it would make the gaining of fully informed consent impossible because the risks would be unknown [31].

### Other options for consent

In light of the concerns regarding the possibility of gaining fully valid informed consent for neonatal and perinatal trials, and yet the generally prevailing views in both the empirical and the analytical research in favour of getting at least some form of consent from parents, analytical research has also discussed other ways of addressing the consent requirement. One claim defends the idea of a waiver of consent in emergencies [18, 36–38], but to this view can then be added (all or some of) a series of constraints. Thus, it is suggested that consent can be waived, but that provisional assent must be given at the time of being invited to participate in the trial [37] and that there should be community involvement at the design stage for the trial protocol [30]. It is claimed that this waiver procedure would not violate the principle of autonomy [30]. However, the aim must be to make research possible for the benefit of patients, and this waiver process should not be used to make research easier for researchers [30].

A second approach defends a method by which patients’ consent is achieved through giving them antenatal notification of the intended clinical trial and then seeking consent if they meet the criteria for entry into the trial [36, 38]. This process is purported to have the advantages of (1) allowing the potential subject extra time to reflect on the information and arrive at a decision [24], thus (2) aiding understanding and (3) reducing anxiety [36]. However, its disadvantages include the facts that (1) there may be logistical problems in delivering the necessary information in this way [30, 37], (2) it places an unnecessary burden on those who will not be eligible [34, 37, 38], and (3) parents may not listen properly if they assume they may not be the ones who will be affected [34]. Proponents of this view claim that these disadvantages can be mitigated by good communication skills [34].

A third option discussed within the analytical research is that of deferred or continuing consent [18, 24, 37]. This is a process in which, if the parents are absent or are affected by situational incapacity, they are assumed to give initial consent and then actually provide full consent when they are capable of taking in the information and making the decision [18]. There is an important objection to this suggestion, and this is that in it no one actually consents to enrolment [18, 24], which also leaves open the possibility that the subject will subsequently not consent, in which case the trial intervention would have constituted assault.

However, full consent provides only ‘permission to continue’. It cannot provide retrospective consent [18, 36]. Furthermore, such subsequent full consent is irrelevant with regard to one-off interventions [30, 36] because it becomes redundant [18] (although it is still applicable to use of personal data for research).

The concept of retrospective consent is considered in the literature. However, this method is argued to be ‘logically incongruent’ [18]. Consent is not actually given, so that, once again, if consent is retrospectively withheld, the researcher is left in the position of never having had consent and thus can be accused of having engaged in assault on the subject.

Another option is the Zelen method of gaining consent [12, 39]. In this approach, parents are not informed if the novel ‘intervention’ will not be offered to the child [40, 41]. Thus, it is argued that this avoids any additional distress [41] which arises for the parents, either from participating in the consent process itself [12, 40] or from knowing that they are in the control group [40]. But, it is argued, this method still respects the rights of the family to know what is happening [12, 39]. One might doubt whether this method deals fully with the challenges for parents to achieve adequate understanding, noted in the section on validity of consent above. However, other concerns are also raised, namely that there are statistical or methodological problems with the method [35] and that the method really circumvents the consent requirement rather than addressing it [35].

The final suggestion is the opt-out method for giving consent. In this case, as the name suggests, the subject is assumed to consent unless she or he explicitly opts out of the trial. The advantages of this process are said to be that it may lessen the burden on parents, it may increase recruitment, and it may increase understanding [34]. However, the objections are that autonomy may be overridden in such a process, such as if the subject fails to exercise her opt-out right by default rather than really opting out autonomously [34].

Defenders of this final proposed alternative method suggest that this problem could be minimised by good communication [34]. It is also argued that the loss of autonomy at that stage (i.e., at the time of opting out) could be ‘offset’ by continued discussion after the event. This would ‘restore autonomy’ by giving more time for information processing [34]. It has also been suggested that a mixed method in which the potential subject is (1) provided with antenatal notification, then (2) given the right to opt out of the research (but assumed to opt in if she or he does not exercise that right), and (3) engaged in a further process of continuing consent amounts as a whole to a procedure of attaining ‘presumed consent’ [34].

Clearly, all these approaches involve a parent giving (or waiving) consent, but a final alternative is to have a consent process in which an independent proxy gives consent for the child. However, it is claimed, against this process of seeking consent, that it is difficult to find a truly independent proxy [18]. It also is argued that there is a need for a truly independent proxy to ensure that the interests of potential trial participants are not conflated with the interests of the research team or of the wider society, or for that matter of some other wider group such as the subject’s family [18]. It is also suggested that this form of consent does not ‘feel’ as if it honours autonomy in the same way as genuine consent does [18].

## Discussion

What light, then, does the analytical literature cast on an ethically defensible approach to obtaining informed consent in perinatal clinical trials? We suggest that these results reveal five key points about the consent process and highlight one important gap in the research. The key points are as follows:

- There are a variety of possible defences for seeking parental ‘consent’ to neonatal and/or perinatal clinical trials, and these are consistent with the strongly and widely held view that it is important that parents do give (or decline) consent for such research, as found in the empirical literature [1].
- In giving parental ‘consent’ in a perinatal context, parents are authorising infant participation, not giving ‘proxy consent’.
- There are philosophical reasons for supposing that at least some parents will fail to give valid consent in a neonatal context. These support concerns about the consent process that are raised in the empirical literature.
- None of the existing consent processes reviewed by the research is satisfactory. This matches the findings of the empirical research.
- There are reasons for giving weight to both parental ‘consent’ and the infant’s best interests in both research and clinical treatment, but also reasons to treat these factors differently in the two contexts, and this may be partly due to the differing relevance of risk in each case.

There is also a significant gap in the philosophical literature, namely a lack of any detailed discussion of a process of emergency and/or urgent assent followed by later full consent, matching a gap found in the empirical literature [1].

Now we take each key point in turn. There has been considerable discussion over a number of years of the justification for seeking parental consent to perinatal and/or neonatal clinical trials. Firstly, the consensus of argument suggests that this requirement is justified, but there is not definite agreement on a single best account of the justification. Secondly, if there is justification for parental involvement, what is given does not count as direct consent, because the child is not expressing a view. Furthermore, the parental involvement is better understood in relation to an analysis of the nature of parenting in which the parent is permitting or authorising the child’s participation, than as the parent giving proxy consent.

This second point relies on the idea that parenting brings with it certain rights or duties, a responsibility to take certain decisions about the child’s treatment. This might be seen as a simple duty, not as requiring further defence or, writers suggest, as something to be further defended (1) in terms of the parental role in relation to the child’s best interests or (2) in terms of the fact that the parents will have to deal with the consequences of the way in which the child is treated. By contrast, whilst a notion of proxy consent may apply in at least some circumstances where a child is not competent to consent, in this case the child has not yet formed relevant beliefs and values on the basis of which a proxy decision could be made.

If, then, in the neonatal context, parents should be seen not as giving ‘proxy’ consent but as authorising or giving permission, how is this related to pursuit of the child’s best interests? One argument, above, is that parental ‘authorisation’ is justified because parents have a special relation to the child’s best interests. However, whilst there might be something in this, it is also noted that in research the researcher and ethics review process must take primary responsibility for the best interests of subjects, so the parental contribution to protecting the child’s interests should be, at most, secondary.

Given the problems parents often have in giving valid consent in the context of neonatal clinical trials, why might not the whole process of parental authorisation be dispensed with and the issue considered entirely in terms of the child’s best interests? Here the intersection with the empirical literature appears particularly important. For the view of parental authorisation as in some way part of a parent’s role coheres well with the widely held view amongst parents that they should be consulted, even if the process of consultation is imperfect by the standards of ‘fully informed consent’. This supports the need for a process which gives space to parental involvement on behalf of the child.

Closely connected to this issue is our third claim, which questions the validity of the consent process in neonatal research. The research here provides arguments explaining why a range of factors (e.g., temporal issues, emotional disturbance or coercive circumstances) all make an impaired consent process likely in this context. This complements the empirical findings that concerns about the consent process are warranted.

Our fourth point is equally consistent with those findings. Although a variety of consent processes have been considered or tried, there are reasons to find fault in each of them. One response to this would be to suggest that the weight given to consent in the research process (as well as to best interests) should not differ from that given to it in the clinical context, and this might allow it to be dispensed with in certain circumstances. Our final substantive conclusion drawn from the analytical literature is that a clinical intervention does differ from offering an infant participation in a clinical trial, and this may be due to the role of risk in research. Thus, once again, even if the process is imperfect, there is still reason to seek parental consent for research.

Finally, there is one significant gap in the analytical research. This gap concerns discussion of a new process for seeking parental authorisation, one which acknowledges that the circumstances of neonatal clinical trials make fully informed consent at the outset impossible, but which seeks to make the best room possible for parental involvement. Considered reflection needs to be given to this process, in which parents assent to or refuse their baby’s participation as best they can during the emergency and later give full consent to continuing participation and follow-up.

## Conclusions

Philosophical analysis supports empirical research in several claims. It notes that it is important that parental ‘consent’ be sought for neonatal research participation, but suggest this is better understood as parental ‘authorisation’ of the participation. However, the analysis also shows that this should be at best a secondary way of protecting the child’s best interests. Furthermore, the analysis gives reasons why at least some parents will fail to give valid consent in a neonatal context, as no existing consent process has been found satisfactory. However, little detailed philosophical attention has been paid to a possible new process for seeking parental authorisation, namely emergency and/or urgent assent followed by later full consent.

## Additional files

Additional file 1: Search strategy. Databases searched and example search strategy. (PDF 213 kb) Additional file 2: Preferred Reporting Items for Systematic Reviews and Meta-Analyses (PRISMA) flow diagram. Diagrammatic representation of the study flow through the review process. (PDF 178 kb) Additional file 3: Full tabulated results. Full findings from all the papers included. (The findings discussed in the Results section are those which contributed to the broader theme particularly relevant to our research question.) (PDF 268 kb)

## Footnotes

1 In philosophical work, this is sometimes arrived at by using a mind-mapping technique.

## References

[1] Wilman E, Megone C, Oliver S, Duley L, Gyte G, Wright J (2015). The ethical issues regarding consent to clinical trials with pre-term or sick neonates: a systematic review (framework synthesis) of the empirical research. Trials.

[2] Coverdale JH, McCullough LB, Chervenak FA (2008). The ethics of randomized placebo-controlled trials of antidepressants with pregnant women: a systematic review. Obstet Gynecol.

[3] Strech D, Synofzik M, Marckmann G (2008). How physicians allocate scarce resources at the bedside: a systematic review of qualitative studies. J Med Philos.

[4] Strech D, Synofzik M, Marckmann G (2008). Systematic reviews of empirical bioethics. J Med Ethics.

[5] Ross D, translator (1980). Aristotle: the Nicomachean ethics.

[6] Waterfield R, translator (1996). Aristotle: physics.

[7] Thomas J, Harden A, Newton M, Gough E, Oliver S, Thomas J (2012). Synthesis: combining results systematically and appropriately. An introduction to systematic reviews.

[8] Faden RR, Beauchamp TL, King NM (1986). A history and theory of informed consent.

[9] Mason SA, Megone C (2001). European neonatal research: consent, ethics committees and law.

[10] Droste S, Dintsios CM, Gerber A (2010). Information on ethical issues in health technology assessment: how and where to find them. Int J Technol Assess Health Care.

[11] Fangerau H (2004). Finding European bioethical literature: an evaluation of the leading abstracting and indexing services. J Med Ethics.

[12] Your baby is in a trial. Lancet. 1995;345(8953):805–6. 7898222

[13] Rostain AL, Bhutani VK (1989). Ethical dilemmas of neonatal–perinatal surgery. Clin Perinatol.

[14] McDonnell K (1990). Volunteering children. Proc Am Cathol Philos Assoc.

[15] Schneider CE (1988). Rights discourse and neonatal euthanasia. Calif Law Rev.

[16] Chervenak FA, McCullough LB (2003). Ethics of research and the pregnant patient. Curr Womens Health Rep.

[17] Kodish E (2003). Informed consent for pediatric research: is it really possible?. J Pediatr.

[18] Brierley J, Larcher V (2011). Emergency research in children: options for ethical recruitment. J Med Ethics.

[19] Dimichele DM (2008). Ethical considerations in clinical investigation: exploring relevance in haemophilia research. Haemophilia.

[20] Pinkerton JV, Finnerty JJ, Lombardo PA, Rorty MV, Chapple H, Boyle RJ (1997). Parental rights at the birth of a near-viable infant: conflicting perspectives. Am J Obstet Gynecol.

[21] DeGrazia D (1995). Value theory and the best interests standard. Bioethics.

[22] The perils of paediatric research. Lancet. 1999;353(9154):685. 10073503

[23] Gamble SA (1996). The ethics of research in the neonatal intensive care unit. Nurs Prax N Z.

[24] Manning DJ (2000). Presumed consent in emergency neonatal research. J Med Ethics.

[25] Goering S (2009). Postnatal reproductive autonomy: promoting relational autonomy and self-trust in new parents. Bioethics.

[26] American College of Obstetricians and Gynecologists, Committee on Ethics; American Academy of Pediatrics, Committee on Bioethics (2011). Maternal-fetal intervention and fetal care centers. Pediatrics.

[27] Franck LS (2005). Research with newborn participants: doing the right research and doing it right. J Perinat Neonatal Nurs.

[28] Jones S, McGee P (2007). Ethical issues in researching pregnant women: a commentary. Res Ethics.

[29] Silverman WA (1988). SSPR mini-symposium: methodologic controversies in clinical research: consent for experimentation involving neonates. Am J Med Sci.

[30] Cuttini M (2004). Intrapartum prevention of meconium aspiration syndrome. Lancet.

[31] Vain N, Prudent L, Szyld E, Wiswell T (2004). A difficult ethics issue. Lancet.

[32] Tyson JE, Knudson PL (2000). Views of neonatologists and parents on consent for clinical trials. Lancet.

[33] Allmark P, Mason S (2006). Should desperate volunteers be included in randomised controlled trials?. J Med Ethics.

[34] Lantos JD (1999). The “inclusion benefit” in clinical trials. J Pediatr.

[35] Silverman W, Altman D (1996). Patients’ preferences and randomised trials. Lancet.

[36] Amin SB, McDermott MP, Shamoo AE (2008). Clinical trials of drugs used off-label in neonates: ethical issues and alternative study designs. Account Res.

[37] Boyle RJ, McIntosh N (2001). Ethical considerations in neonatal resuscitation: clinical and research issues. Semin Neonatol.

[38] Vain NE, Szyld EG, Prudent LM, Wiswell TE, Aguilar AM, Vivas NI (2004). Oropharyngeal and nasopharyngeal suctioning of meconium-stained neonates before delivery of their shoulders: multicentre, randomised controlled trial. Lancet.

[39] Worrall J (2008). Evidence and ethics in medicine. Perspect Biol Med.

[40] Allmark P (1999). Should Zelen pre-randomised consent designs be used in some neonatal trials?. J Med Ethics.

[41] Braunholtz DA (1999). A note on Zelen randomization: attitudes of parents participating in a neonatal clinical trial. Control Clin Trials.

