# Supplementary material for: The ethical issues regarding consent to clinical trials with pre-term or sick neonates: a systematic review (framework synthesis) of the analytical (theoretical/philosophical) research
Source: Trials. 2016 Sep 9;17(1):443. doi: 10.1186/s13063-016-1562-3 (PMC5016881; doi:10.1186/s13063-016-1562-3)
Supplement: Additional file 1: — Search strategy. Databases searched and example search strategy. (PDF 213 kb) [file 13063_2016_1562_MOESM1_ESM.pdf]

Additional file 1 Search strategy

First draft searches were developed in July 2011 using MEDLINE (Ovid) and Philosopher's Index (Ovid). The emerging literature helped to develop the initial conceptual framework further. The second searches were devised in October 2011 with attention to the confirmed framework, terms suggested by the project team, database index terms, and recent literature listing terms useful in retrieving bioethics studies [4,10].

Book catalogues were included due to the importance of book materials as a source of philosophical works.

Electronic resources for the second searches (October 2011) and 'update' searches (January 2014)

- BIOSIS Previews <1969 to 2014 Week 09>
- Embase Classic+Embase (Ovid) <1947 to 2014 January 29>
- Ovid MEDLINE(R) <1946 to January Week 3 2014>
- Ovid MEDLINE(R) In-Process & Other Non-Indexed Citations <January 29, 2014>
- The Philosopher's Index <1940 to September 2013>
- PsycINFO <1806 to January Week 3 2014>
- CINAHL (EbscoHost) 1981 - present
- Social Science Citation Index (Web of Science, Thomson Reuters) 1898 – present
- Social Services Abstracts (ProQuest - CSA) 1979-present
- University of Leeds Library Catalogue
- WorldCAT (OCLC)

Example Search Strategy

Ovid MEDLINE(R) <1946 to January Week 3 2014>

1 Infant, Premature/ (39793)

2 exp infant, premature, diseases/ (35913)

3 exp infant, newborn, diseases/ (138575)

4 neonatology/ (2016)

5 perinatology/ (1605)

6 neonat*.tw. (180115)

7 (preterm adj3 (baby or babies or infant* or deliver* or birth*)).tw. (29863)

8 (pre-term adj3 (baby or babies or infant* or deliver* or birth*)).tw. (1134)

9 (premature adj3 (baby or babies or infant* or deliver* or birth*)).tw. (23017)

10 (pre-mature adj3 (baby or babies or infant* or deliver* or birth*)).tw. (12)

11 ((disease* or illness* or sickness*) adj3 (newborn or "new born" or baby or babies* or infant*)).tw. (9647)

12 ("intensive care" adj3 (newborn or "new born" or baby or babies* or infant*)).tw. (1459)

13 ("Critical Care" adj3 (newborn or "new born" or baby or babies* or infant*)).tw. (28)

14 (perinat* or "feto-maternal*").tw. (50661)

15 or/1-14 (357828)

16 (ethic* or moral* or bioethic*).tw. (90452)

17 (integrity adj4 (body or bodily or physical or medical)).tw. (1004)

18 (integrity adj2 (professional or physician* or female or male or child* or parent* or personal or family)).tw. (500)

19 (beauchamp adj3 childress).tw. (57)

20 ("social shaping" or casuistry or "right to die" or "right to life").tw. (1534)

21 (ELSI or rawls or rawlsian).tw. (210)

22 (Conflict* adj2 interest*).tw. (3886)

23 (complicit* or humanism or dignity or human right* or principlism or principle-base* or beneficence).tw. (11812)

24 (patient* adj1 autonomy).tw. (1754)

25 (parent* adj4 autonomy).tw. (265)

26 justice.ti. (2941)

27 fairness.ti. (467)

28 equity.ti. (2056)

29 access.ti. (24950)

30 harm.ti. (4059)

31 freedom.ti. (2993)

32 prejudice.tw. (2496)

33 philosoph*.tw. (19372)

34 (non-maleficence or aristoteles or Socrates or Socratic or "reflective equilibrium*").tw. (592)

35 (value* adj2 (personal or professional or social or society or societal or ethnic or judgement*)).tw. (3490)

36 altruis*.tw. (2806)

37 (public* adj2 opinion*).tw. (1699)

38 (legal* adj2 issue*).tw. (2719)

39 socio-cultural context*.tw. (243)

40 "quality of life".ti. (35132)

41 (attitude* adj2 (parent* or physician* or consultant* or nurse* or doctor* or mother* or father* or midwife*)).tw. (6314)

42 (view* adj2 (parent* or physician* or consultant* or nurse* or doctor* or mother* or father* or midwife*)).tw. (3708)

43 or/16-42 (212333)

44 morals/ or conscience/ or ethics/ or moral development/ or virtues/ (20803)

45 ethics.fs. (47461)

46 ethics/ or bioethical issues/ or bioethics/ or ethics, clinical/ or exp "codes of ethics"/ or complicity/ or "conflict of interest"/ or ethics, professional/ or ethics, medical/ or hippocratic oath/ or therapeutic equipoise/ or ethics, nursing/ (83232)

47 *prejudice/ or *social values/ (16732)

48 "attitude of health personnel"/ or attitude to death/ or attitude to health/ or health knowledge, attitudes, practice/ (217736)

49 Altruism/ (4952)

50 principle-based ethics/ or beneficence/ or personal autonomy/ or social justice/ or professional autonomy/ (30712)

51 personhood/ (3088)

52 *"quality of life"/ or *"value of life"/ (51286)

53 freedom/ (5032)

54 disclosure/ or mandatory reporting/ or parental notification/ or truth disclosure/ or duty to warn/ or whistleblowing/ (25276)

55 or/44-54 (426796)

56 43 or 55 (525224)

57 Informed Consent/ (30594)

58 Parental Consent/ (2871)

59 decision making/ or choice behavior/ or consensus/ or "dissent and disputes"/ or refusal to participate/ (91270)

60 negotiating/ or uncertainty/ (10432)

61 Research Subjects/ (4832)

62 human experimentation/ or therapeutic human experimentation/ (11035)

63 (consent* adj2 (informed or parental*)).tw. (22222)

64 (parent* adj2 (choice* or decision* or decide* or choose* or prefer* or permission*)).tw. (2309)

65 (informed* adj2 (choice* or decision* or preference*)).tw. (5654)

66 ((Research or trial) adj3 (subject or subjects or volunteer or volunteers or participa*)).tw. (22420)

67 or/57-63 (150000)

68 (empirical* adj3 (analys* or study or studies or design* or method*)).tw. (11934)

69 (analytical* adj3 (analys* or study or studies or design* or method*)).tw. (23543)

70 (philosoph* adj3 (analys* or study or studies or design* or method* or argue* or discuss*)).tw. (1313)

71 exp ethical analysis/ or exp "ethical review"/ or ethical theory/ or ethics, research/ (12564)

72 ((trial* or study) adj3 (design* or recruitment* or participat* or method*)).tw. (398005)

73 exp clinical trials as topic/ (271469)

74 pilot projects/ (79068)

75 feasibility studies/ (41016)

76 intervention studies/ (6287)

77 or/68-76 (805296)

78 15 and 56 and 67 and 77 (226)
