# Supplementary figures and images for: The ethical issues regarding consent to clinical trials with pre-term or sick neonates: a systematic review (framework synthesis) of the analytical (theoretical/philosophical) research
Source: Trials. 2016 Sep 9;17(1):443. doi: 10.1186/s13063-016-1562-3 (PMC5016881; doi:10.1186/s13063-016-1562-3)

**Additional file 2 PRISMA Flow Diagram for analytic papers**

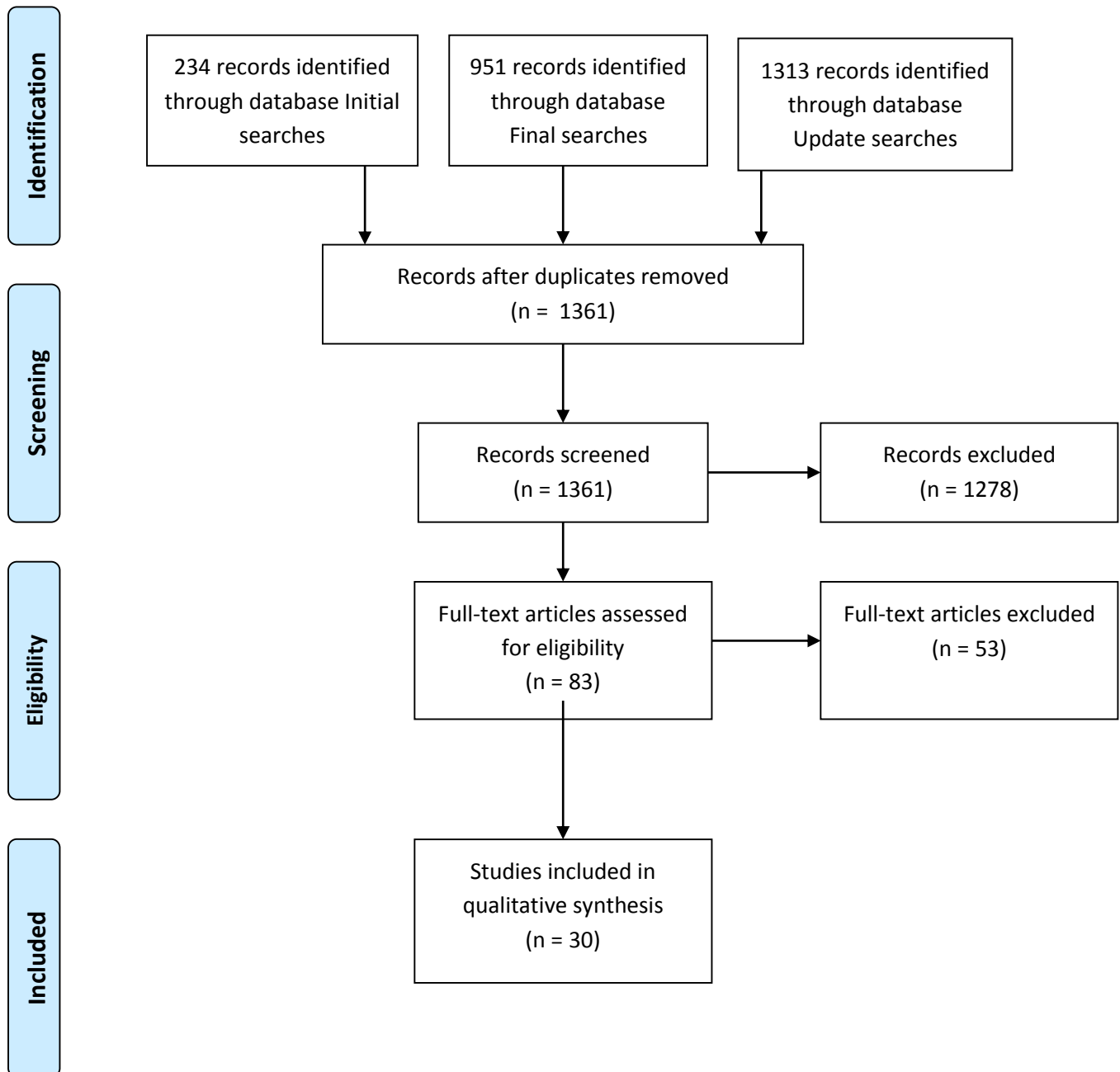

Supplement: Additional file 2: — Preferred Reporting Items for Systematic Reviews and Meta-Analyses (PRISMA) flow diagram. Diagrammatic representation of the study flow through the review process. (PDF 178 kb) [file 13063_2016_1562_MOESM2_ESM.pdf]
