# Supplementary material for: The ethical issues regarding consent to clinical trials with pre-term or sick neonates: a systematic review (framework synthesis) of the analytical (theoretical/philosophical) research
Source: Trials. 2016 Sep 9;17(1):443. doi: 10.1186/s13063-016-1562-3 (PMC5016881; doi:10.1186/s13063-016-1562-3)
Supplement: Additional file 3: — Full tabulated results. Full findings from all the papers included. (The findings discussed in the Results section are those which contributed to the broader theme particularly relevant to our research question.) (PDF 268 kb) [file 13063_2016_1562_MOESM3_ESM.pdf]

Additional File 3 Full tabulated findings

The ethical basis of parental informed consent for neonatal/perinatal research

Where children are unable to give or decline consent, parents should do so

- Based in autonomy
  - Parental autonomy/parents' own rights (12, 13)
    - Making decisions about a child is part of what it is to be a parent (14)
    - +/- as a 'privacy' right (15)
    - Parenting decisions is part of deciding how to conduct one's own life (13, 15)
    - Fetal rights as a function of maternal autonomy (16)
- Objections
  - Does parents' interest in autonomous parenting outweigh child's interests? (15)
  - Parents are no longer 'owners' of their children' (17)
- Parents as surrogate decision-makers (13)
  - When a child is not yet autonomous the parent is the most appropriate 'proxy' (18)
  - May have knowledge of personality, values and beliefs (older children) (18)
  - Not appropriate to think of 'autonomy' of a neonate (17, 18)
  - 'Family decision-making' – not appropriate to consider the child's decision in isolation (14)
  - Not appropriate to think of 'informed consent' for a child (14)
  - Parents will bear the consequences of the decision (13)
  - Legal 'rights' not appropriate for family law (15)
- Beneficence – purpose of informed consent is to protect best interests of child (12, 17)
  - Responsibility of parents to make decisions as a way of promoting child's best interests (13, 19)

        - Parents most likely to have best interests at heart (15, 20)
        - Parents are best judge of best interests (15, 17, 18, 20)(Objection to this – justification is prudential and therefore insecure (15))
        - Parents should only be allowed to make the decision when it is in the best interests of the child (13, 14, 17, 20)
    - Protection of best interests should not rest entirely on informed consent (12, 17)
- Never ethically obligatory to consent to research (16)
    - Because this would conflict with altruistic motivation (17)
- Determining what is meant by best interests of neonate is not simple (mental state account vs desire-satisfaction account vs objective list account) (21)
- Triangle of paediatric ethics – child on top, parents and clinicians to lend support (17)
- Parental consent is a misnomer – should be parental permission (17)
- Parents (not courts) should make decisions to promote pluralism (15)

The validity of parental consent

Potential barriers (22)

- Time limitation - simply not possible to ask for consent
  - Time limit affects information given (23, 24)
  - Time limit affects understanding (24)
- Stress, anxiety, pain, sedation (23, 24)
  - Affecting understanding (23, 24, 25)
  - Affecting capacity (23, 25)
  - Contributing (positively) to autonomous decision process (13)
- Desperation, fear (23, 24, 26)
  - Affecting voluntariness (15, 24, 25)
  - Does not affect voluntariness if there was no 'right' to receive the unproven treatment (23)
- Consent process increases parental anxiety (24)
- Researcher as a figure of authority/power
  - Affecting voluntariness (28)

Undermining individuals' appreciation of their own competence is an obstacle to autonomy (25)

Implications of barriers to informed consent

- Principle of autonomy
  - Violated if there is a defect in the consent process (24)
  - Parents used as a means to an end in the consent process (24)

Beneficence becomes a more important principle in research with neonates because informed consent is not possible (therefore autonomy/respect for persons should be applied with caution) (27)

Must strive for improvement/best possible consent even where perfect informed consent not possible (17)

Deprived/uneducated parents more likely to consent

- Conclusion cannot be generalised (24, 29)
- Not a just allocation of burden (24, 29)

Risk and double standard/disanalogy/asymmetry between consent for research and consent for clinical treatment

- Ambiguities of risk
  - If context is potentially life-threatening and outcome is unknown this should be viewed as significant risk (30)
  - If context is potentially life-threatening this is a risk of the disease/situation and not the trial, so should not be viewed as a risk of the trial intervention (31)
- Fully informed consent not possible for clinical trials because the very information needed for informed consent is that which is uncertain and under investigation (32)
- Beneficence trumps in clinical decisions (33)
- Autonomy trumps in research decisions (24)
  - Because the two types of consent evolved separately (29)
  - Because equipoise means appeals to beneficence in research are less convincing (13, 16, 17)
- Where there is genuine equipoise therapeutic research is not significantly different from treatment (33)
  - There should be no difference in principles between the two (32, 33)
- If there is inclusion benefit should research be any different to treatment? (34)
- Stricter ethical guidelines in research acknowledge the altered doctor/patient relationship (34)
- RCTs needed to protect patients from the use of untested treatments (23, 35)
  - Using unproven treatments is in itself a violation of autonomy (31)

Other options for gaining consent

Waiver of consent in emergency situations (18, 36, 37, 38)

- With provisional assent at time (37)
- With community involvement at design stage (30)
- Principle of autonomy not violated (30)
- Aim is to make research possible for benefit of patients (30)

Antenatal notification and consent (36, 38)

- Advantages
    - Extra time (24)
        - Aids understanding (36)
        - Reduces anxiety (36)
- Disadvantages
    - Logistical problems (30, 37)
    - Unnecessary burden on those who are not eligible (34, 37, 38)
    - Parents may not listen properly if assuming they may not be affected (34)

    These disadvantages can be mitigated by good communication skills (34)

Deferred/continuing consent (18, 24, 37)

- Full consent when they are capable of taking in the information and making decision (18)
    - ‘Permission to continue’ (18, 36)
    - Irrelevant if the trial is a one off intervention (30, 36)
- Justification for not obtaining initial full consent
    - Parents absent (18)

- Situational incapacity (18)

- Objections
    - No-one actually consents to enrolment (18, 24)

Proxy consent (18)

- Difficult finding truly independent proxy (18)
- Doesn't feel as if it honours autonomy in the same way (18)
- Needs truly independent proxy to ensure that interests of participants are not conflated with interests of research team or society (18)

Zelen method (12, 39)

- No need to inform patients if the 'intervention' does not affect them (39, 41)
- Avoids distress (41)
    - Of consent process itself (12, 40)
    - Of knowing they were in the control group (40)
- Still respects rights of family to know what is happening (12, 39)
- Statistical/methodological problems (35)
- Circumvents consent requirement (35)

Retrospective consent

- Logically incongruent (18)

Opt-out

- Advantages
    - May lessen burden on parents (34)
    - May increase recruitment (34)

    - May increase understanding (34)
- Objections
    - Autonomy overridden (34)
        - If fails to exercise opt-out right by default rather than autonomously (34)
        - This could be minimised by good communication (34)
        - Loss of autonomy at that stage could be ‘offset’ by continued discussion after the event (34)
    - ‘Restore autonomy’ by giving more time for information processing (34)

Antenatal notification + opt-out + continuing consent = ‘presumed consent’ (24, 34)

Miscellaneous themes

- Father has moral interest in health of fetus and pregnant woman (29)
- Fetal interventions have physical risks and implications for pregnant women (29)
    - Consent must be based on pregnant woman's assessment of her own interests (29)
- Pregnant women are subject to advice, influence and pressure from partners/mothers/in-laws/healthcare providers/the media, and it is therefore difficult for a pregnant woman to make her own decisions (28)
